# An Assessment of Behavior Change Techniques in Two Versions of a Dietary Mobile Application: The Change4Life Food Scanner

**DOI:** 10.3389/fpubh.2022.803152

**Published:** 2022-02-23

**Authors:** Sundus Mahdi, Emily K. Michalik-Denny, Nicola J. Buckland

**Affiliations:** ^1^School of Health and Related Research, University of Sheffield, Sheffield, United Kingdom; ^2^Department of Psychology, University of Sheffield, Sheffield, United Kingdom

**Keywords:** Behavior Change Techniques, mobile applications, childhood obesity prevention, diet and nutrition, mHealth, digital intervention

## Abstract

The Change4Life Food Scanner app is a UK Government dietary app designed to provide feedback on the nutritional content of packaged foods to parents and their children. To understand its intended mechanism of behavior change and how Behavior Change Technique (BCT) content evolves with app updates, this research aimed to map out the BCTs of two versions of the Change4Life Food Scanner app. Two coders undertook a descriptive comparative analysis of the use of BCTs in the Food Scanner app using the Behavior Change Technique Taxonomy [both the outdated (v1.6) and updated (v2.0) versions of the app were coded]. Results showed that both versions encompass the BCTs “goal setting (behavior)”, “feedback on behavior”, “social support (unspecified)”, “instruction on how to perform behavior”, “salience of consequences”, “prompts/cues” and “credible source”. The outdated version contained the BCT “behavior substitution” which had been dropped in the updated version. The updated version featured the additional BCTs “information about social and environmental consequences”, “information about emotional consequences”, “social reward” and “social incentive” and was comparatively more BCT intensive in terms of content and occurrence. The BCT content of the Food Scanner app resembles that of existing dietary apps and incorporates several BCTs which have previously been found to be effective. Future work to evaluate the effectiveness of the app is recommended. This will provide insight into whether the combination of BCTs used in the Change4Life Food Scanner app are effective in improving dietary choices.

## Introduction

In the UK, one in three children in the final year of primary school are with overweight or obesity (1). Given that children's diets are heavily dictated by their parents, interventions that target families' nutritional choices may play a key role in preventing and tackling childhood obesity, and reducing the burden of preventable diseases (2).

Smartphone use is popular and provides access to downloadable applications (“apps”). Smartphone applications are self-contained programs that can be accessed easily and are far-reaching, making them a cost-effective and useful method of delivery for behavioral interventions (3). As such, there has been a rise in the development and feasibility testing of app-based interventions targeting childhood obesity prevention through parental behavior change. However, given this area of research is still growing, data on app-effectiveness is limited (4–6), and the majority of app-based interventions are not evidence based (7). Research suggests that interventions with a theoretical basis are more effective in targeting determinants of behavior change (8) and The National Institute for Health and Care Excellence (NICE) guidance advises that behavior change interventions ought to include BCTs which have been found to be effective in changing behavior (9).

A systematic review, investigating the quality of dietary apps targeting children, found that app quality ratings correlated with the presence of BCTs and app features (10). Six BCTs, on average, were identified per app, whereby “providing instructions”, “general encouragement”, “contingent rewards”, and “feedback on performance” were the most frequently adopted. In another review of eleven mobile apps designed to support healthier food purchasing behavior, 1–14 BCTs were identified per app (11). All apps had elements of “goal setting (outcome)” and “self-monitoring of outcomes of behavior”. Yet, some of the most frequently used BCTs are not the most effective (12), and there is limited evidence to support BCT content in apps targeting families. More recently, interventions targeting parents for childhood weight management have considered the Behavior Change Wheel (BCW) in their design to determine the inclusion of evidence based BCTs (13). However, the number of available studies that have included BCT mapping of family-based dietary digital interventions are limited (14) and data on intervention effectiveness is yet to be published (15). In many cases transparency around the use of BCTs goes unreported. Recent NICE guidance has recommended research be conducted to evaluate the specific components and characteristics of digital health interventions, and to what extent they are individually effective at changing behavior (16). Therefore, to know which BCTs are most effective within dietary apps, these apps need to be evaluated in terms of efficacy and BCT content.

The Change4Life Food Scanner app was developed by Public Health England as part of a wider public health campaign to promote healthy lifestyle choices (17). The app targets 5–11 year old children and their parents and has over 500,000 installs on Google Play (18). The app aims to encourage parents to improve their children's dietary intake by promoting healthier food choices. Users can scan the barcode of packaged products and receive feedback about the nutritional content of the item (e.g., through traffic light nutritional labels or sugar cubes, salt sachets or fat slabs to describe quantity). Understanding the BCTs used in the Change4Life Food Scanner app is important to allow for the comparison of BCTs used within various dietary apps. This is essential if the effectiveness of complex interventions that adopt BCTs is to be adequately evaluated and could later help inform the development of effective mHealth interventions.

Although research exists on the range of BCTs currently adopted in dietary mHealth interventions, the majority of these are not focused on child outcomes (10) and are reviews of the BCTs incorporated in a range of dietary apps available on the app market (11, 19, 20). It is unclear which BCTs are related to which apps, and whether these apps have been developed by reliable sources. Additionally, one of the difficulties analyzing app-based interventions is that they are frequently updated, including both content and design features. The Food Scanner app underwent a major update in June 2020 after this research had commenced. Changes to the BCTs used during the lifecycle of app-based interventions could lead to complications in the evaluation process and are therefore important to assess. Therefore, the aim of this research was to map out the BCT content of the Food Scanner app to understand the intervention's intended mechanism of behavior change. Additionally, this research aimed to compare the BCT content of the previous and new versions of the app.

## Methods

### Study Design

A descriptive comparative analysis of the use of BCTs in the outdated (v1.6; March 2016) and updated (v2.0; June 2020) version of the Food Scanner app was undertaken in August 2020. BCTs used for continued app use (app engagement) and encouraging healthy dietary choices were the outcomes of interest. Dietary choices included reference to any food groups and/or macronutrients.

### Coder Training

Two coders undertook an online training program affiliated with the Behavior Change Technique Taxonomy Version 1 (BCTTv1) which consisted of six training sessions and two assessments (required pass rate competency ≥60%) (21). The BCTTv1 is a nomenclature of 93 BCTs clustered into sixteen domains, designed to aid researchers and experts in reporting intervention content (8).

### BCT Mapping

Both coders independently used the updated version of the app until they had accessed all features and were no longer able to generate new outputs from the app (“data saturation”) (22). The coders then independently mapped the BCT content of the app using the BCTTv1. Mapping involved recording “evidence” of each BCT as it occurred. Results were compared between both coders in a discrepancy discussion and a consensus was reached. Within the discrepancy discussion, coders voiced uncertainty about the presence of a few BCTs, whereby the evidence was insufficient to formally code the presence of a BCT (i.e., where the presence of a potential BCT did not fully match the description provided in the BCTTv1). In such cases, the term “near-misses” was applied. Identifying “near-misses” could help to identify areas of the app which could be modified to strengthen the effect of the intervention by fully delivering the near-missed BCTs. In addition to mapping out BCTs from the app directly, the coders researched both versions of the app online to gain a deeper understanding of the apps' intended purposes and features. This included reviewing the app descriptions provided on Google Play and the Apple app store, as well as reviewing any app demonstrations on YouTube. This was undertaken as a validity check to ensure that no app features had been overlooked during app use and testing. In cases where an app feature discussed online was not identified despite extensive app use, the underlying BCT was mapped as a “near-miss”.

The first coder (SM) mapped the outdated version by directly using the app. The second coder (EMD) used secondary evidence that was available online, as at the time of mapping the outdated version was no longer available. The secondary evidence for the outdated version was verified by the first coder given their previous exposure and use of the outdated version of the app. This included app descriptions, video tutorials, screenshots of features, and evidence descriptions provided by the first coder (first coder's BCT findings removed). Both coders mapped the updated version by using the app. Inter-rater reliability was assessed using Kappa. Each coder indicated whether each of the 93 BCTs in the taxonomy were present in the outdated and updated versions of the app. This data was entered into SPSS Statistics (version 25) and a Kappa score was calculated for each version of the app. For the outdated version, the Kappa score was 0.94 and for the updated version was 0.89. Both of these Kappa scores are indicative of very good agreement (23). As part of the mapping exercise, coders documented BCT presence, the features of the app where BCTs were present, the frequency of each BCT presence, and the average occurrence of each BCT. A Pearson Chi-Square test of independence was also undertaken to compare BCT presence between app versions.

## Results

### Outdated Version (v1.6)

Eight out of ninety-three BCTs (8.6%) were identified including “goal setting behavior”, “feedback on behavior”, “social support (unspecified)”, “instruction on how to perform the behavior”, “salience of consequences”, “prompts/cues”, “behavior substitution” and “credible source”. These BCTs belong to eight of sixteen domains (50%) including “goals and planning”, “feedback and monitoring”, “social support”, “shaping knowledge”, “natural consequences”, “associations”, “repetition and substitution” and “comparison of outcomes”. On average, each BCT appeared in 2.5 different features of the app. The most frequent BCT was “feedback on behavior” which involves monitoring behavior and providing informative or evaluative feedback on the performance of the targeted behavior. Feedback occurred through the use of traffic light labels, the visual depiction of sugar/fat/salt content, calorie information, traffic lights and written feedback on scans. The second most frequently occurring BCT was “social support (unspecified)” which was delivered through signposting to further information and through the provision of encouragement in response to scanning items that were considered to be a healthy choice (see Supplementary Table 1 for the mapping results and all available evidence of where BCTs were present).

### Updated Version (v2.0)

Eleven of ninety three BCTs (11.8%) were identified including “goal setting behavior”, “feedback on behavior”, “social support (unspecified)”, “instruction on how to perform the behavior”, “salience of consequences”, “information about social and environmental consequences”, “information about emotional consequences”, “prompts/cues”, “credible source”, “social reward” and “social incentive”. These BCTs belong to eight of sixteen domains (50%) including “goals and planning”, “feedback and monitoring”, “social support”, “shaping knowledge”, “natural consequences”, “associations”, “repetition and substitution”, “comparison of outcomes” and “reward and threat”. On average, each BCT appeared in 2.7 different features of the app. The most frequently occurring BCT was “feedback on behavior” which had several modes of delivery including “low badges”, “woah badges” and a virtual reality feedback feature. The second most frequent BCT was “instruction on how to perform behavior” which was present in the instructional section of the app.

### Comparison of Outdated and Updated Versions

**Figure 1:**
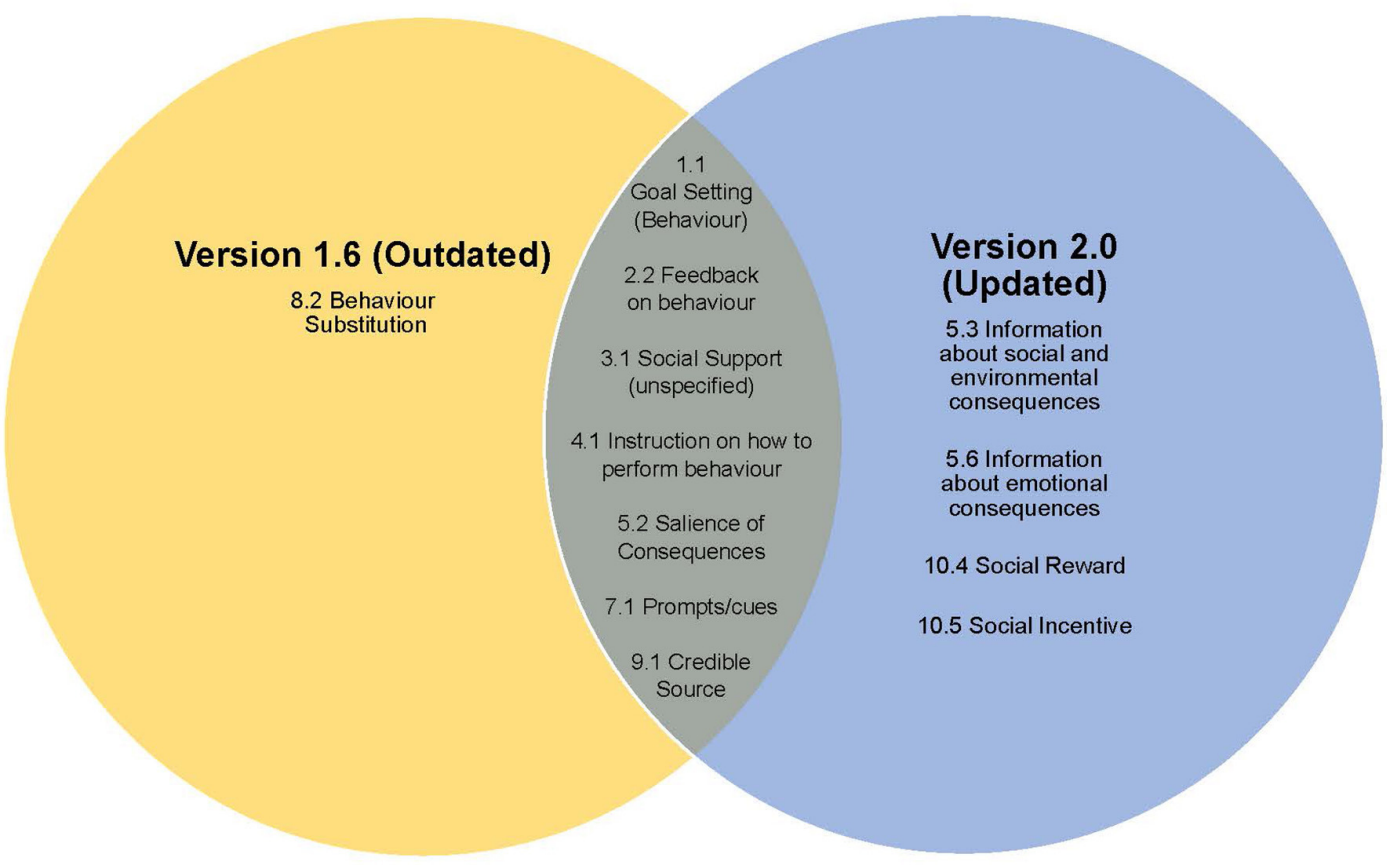
Venn diagram displaying the BCT commonalities and differences between the outdated and updated versions of the Change4Life Food Scanner App.

Figure 1 displays the commonalities and differences between the two versions of the app. The updated version had a significantly greater BCT presence than the outdated version of the app [*X*^2^ (1, *N* = 93) = 48.06, *p* < 0.001]. The updated version included three more BCTs than the outdated version and had a higher mean occurrence of each BCT (see Table 1). Although each version comprised of BCTs from eight of the BCTTv1 taxonomy domains, the outdated version included a BCT from “repetition and substitution” while the updated version included BCTs from “reward and threat”. The outdated version of the app incorporated the BCT “behavioral substitution”, however there was no evidence of this BCT in the updated version. Furthermore, the updated version was found to include the BCTs “information about social and environmental consequences”, “information about emotional consequences”, “social reward” and “social incentive” which were not present in the outdated version of the app. There was a comparatively higher emphasis on the domain of “natural consequences” in the updated version while the outdated version focused more on “social support”. While the BCT “salience of consequences” was delivered in both versions of the app by the visual depiction of salt, fat and sugar content in the form of salt sachets, fat lumps and sugar cubes, the updated version also incorporated a virtual reality and animation element. This provided the user with a 3D image imposed onto the camera view of their device, bringing to life the nutritional content.

**Table 1:** A comparison of Behavior Change Techniques (BCTs) in the outdated (v1.6) and updated (v2.0) versions of the Food Scanner app.

**Code, BCT label and domain**	**Version of Change4Life Food Scanner App**			
	**Outdated version (v1.6)**		**Updated version (v2.0)**	
	**BCT present**	**No. of occurrences of BCT**	**BCT present**	**No. of occurrences of BCT**
1.1 Goal Setting Behavior *Goals and Planning*	✓	2	✓	1
2.2 Feedback of Behavior *Feedback and Monitoring*	✓	7	✓	7
3.1 Social Support (Unspecified) *Social Support*	✓	3	✓	1
4.1 Instruction on how to perform the behavior^*^ Shaping Knowledge	✓	2	✓	5
5.2 Salience of Consequences *Natural Consequences*	✓	1	✓	2
5.3 Information about social and environmental consequences *Natural Consequences*	X	0	✓	2
5.6 Information about emotional consequences *Natural Consequences*	X	X	✓	2
7.1 Prompts/cues^†^ *Associations*	✓	2	✓	3
8.2 Behavior Substitution *Repetition and Substitution*	✓	2	X	0
9.1 Credible Source *Comparison of Outcomes*	✓	1	✓	1
10.4 Social Reward *Reward and Threat*	X	0		2
10.5 Social Incentive *Reward and Threat*	X	0		1
**Total Number of BCTs present**	8	–	11	–
**Mean occurrence of each BCT**	–	2.5	–	2.7

*NB. BCTs have been coded for both app engagement and improved dietary choices*.

* *BCT targeted app engagement only*;

† *BCT targeted dietary choices only; ✓BCT present; X BCT absent*.

Across both versions, most of the BCTs coded were designed to instigate both app engagement (through scanning barcodes) and healthier dietary choices, with the exception of “instruction on how to perform behavior” which targeted app engagement only, and “prompts/cues” which targeted dietary choices only.

### Near-Misses

For the outdated version, coders rated “information about social and environmental consequences” as a near-miss. This related to phrases such as “Woohoo! This choice makes a great start to the day.” Although the language used indicates approval, it was not clear that such a phrase was related to approval of the target behavior, a pre-requisite for coding this BCT.

For the updated version, “social reward” was coded as a near-miss and referred to the “Good Choice” badge feature of the app. The presence of a “Good Choice” badge was described on the Food Scanner app store and online. However, it was coded as a near miss because the badge was not displayed while using the app (despite extensive app use).

Across both app versions, “behavioral practice” was considered a near-miss. “Behavioral practice” referred to a feature where users are prompted to scan barcodes of packaged products. However, it was not clear that the feature explicitly prompted practice in a context where the performance is necessary.

## Discussion

Both versions of the Change4Life Food Scanner app used a small proportion of the total number of BCTs within the BCTTv1. The outdated version used 8.6% and the updated version used 11.8%. Across both app versions, the BCTs “goal setting (behavior)”, “feedback on behavior”, “social support (unspecified)”, “instruction on how to perform behavior”, “salience of consequences”, “prompts/cues”, “credible source”, “behavioral substitution”, “information about social and environmental consequences”, “information about emotional consequences”, “social reward” and “social incentive” were found to be present. The updated version of the app was comparatively more BCT intensive in terms of content and occurrence and had a higher focus on the domain “natural consequences”, adopting three BCTs from this domain, whereby the outdated version only encompassed one BCT from this domain.

The BCT content of the Food Scanner app aligns with similar research that has investigated BCT presence in dietary interventions and includes effective BCTs. BCTs from the domains “goals and planning”, “feedback and monitoring”, “shaping knowledge” and “social support” have been found to be common components of dietary interventions (10, 11, 20, 24). These BCTs (with the exception of “shaping knowledge”) have been outlined within NICE guidance as effective strategies for changing behavior (9). Of the BCTs used in the Food Scanner app, 6/8 (75%) BCTs in the outdated version and 8/11 (73%) BCTs in the updated version have been found to have an effectiveness ratio of 50% or greater in similar interventions (25–27). Other BCTs were included that have also been used in previous research but have limited evidence for their effectiveness [“social incentive” (updated app version), “instruction on how to perform behavior” and “credible source” (both versions)] (25).

Although, the updated version of the Food Scanner app includes more BCTs than the outdated version, the outdated version had a greater percentage of BCTs that have been found to be effective (25–27). The outdated version of the app included the BCT “behavioral substitution”, however, this BCT was removed in the updated version. Evidence suggests that “behavioral substitution” has a high effectiveness ratio in dietary interventions (26), suggesting that the app update removed a potentially effective BCT. There are however other indicators of intervention effectiveness. For instance, the updated version had a greater number of BCT occurrences in comparison to the outdated version. A previous study found a positive correlation between BCT frequency and intervention effectiveness indicating that the update could improve the efficacy of the Food Scanner app (19). These findings contrast with a systematic review which found no association between the number of BCTs and intervention effectiveness (20). Given the contradictory evidence, further research is needed to investigate the association between BCT prevalence and intervention effectiveness.

The Food Scanner app, particularly the updated version, has a strong focus on “natural consequences” and “feedback”, delivering BCTs from these domains in several ways. BCTs “salience of consequences” and “feedback on behavior” have been found to have effectiveness ratios of 83 and 52%, respectively (25–27). Evidence suggests interventions that have a narrow BCT focus (contain several BCTs from the same domain) tend to be more effective, further indicating that the updated version of app possesses a feature of an effective intervention (20, 28, 29). While both versions deliver the BCT “salience of consequences” through the visual depiction of nutritional content in the form of salt sachets, fat slabs and sugar cubes, the updated version incorporates a 3D and animation element to the delivery. This emphasizes the consequences of consuming nutrient poor food in an innovative way making the mechanism of delivery of this BCT more prominent in the updated version. Additionally, while the updated version of the app incorporates “information about social and environmental consequences”, this BCT has been found to have a Non-effective ratio of 100% in interventions to tackle childhood obesity indicating that the app contains at least one BCT that may be ineffective in this setting (27). However, evidence suggests that inclusion of some ineffective BCTs does not have a detrimental impact on an intervention's overall effectiveness (20). Given that “information about social and environmental consequences” has not previously been found to be an effective BCT, providing information about the health consequences instead may be an alternative solution. “Information about health consequences” has been found to be an effective BCT in improving diets of children through parental behavior change (14) and young adults with a 100% effectiveness ratio, and is one that is recommended for use in interventions with the same setting as the Food Scanner app (26, 27).

The coders noted incidences of near-misses where a BCT may have been present but was not coded due to a lack of evidence. This included “information about social and environmental consequences” (outdated version). Although this BCT has previously been found to have a 100% Non-effectiveness ratio (27), its use has been advised through the use of the BCW within similar interventions (15). Although “social reward” was mapped within the updated version of the app, its presence could have been amplified thus potentially strengthening the impact of this BCT, given it has previously been reported to have a 57% effectiveness ratio (25). “Behavioral practice” was considered a near-miss in both versions of the app. Adjustment of the feature to prompt barcode scanning to explicitly prompt the practice of choosing healthier alternatives, could potentially improve the app's effectiveness, given that this has been found to have a 100% effectiveness ratio in similar settings (27). Its inclusion within similar interventions has also been advised (15, 30). Strengthening the content of the Food Scanner app could help increase BCT presence, and potential app effectiveness.

The effectiveness of BCTs adopted within interventions may depend upon the recipient. Therefore, caution should be taken when comparing results to previous studies. Although the Food Scanner app has been designed to improve dietary outcomes of primary school-aged children, the intervention will most likely be received by the parent. The healthiness of the home environment and decisions over what to feed their child will depend upon changes in parental behavior. The app could also be seen as a “shared” intervention, whereby the parent engages the child and decisions are made collectively. Therefore, the use of BCTs within existing studies may not be fully applicable to the Food Scanner app. More recently, mHealth interventions targeting parents have used the BCW Framework to guide the inclusion of BCTs. The SWAP IT trial, which was found to be effective in reducing energy content of packed lunchboxes, integrated six BCTs, including “provision of information about health consequences”, “action planning”, “demonstration of behavior”, “adding objects to the environment”, “prompts and cues”, and “instruction on how to perform the behavior”(14, 31). Of these, only the latter two BCTs were identified within both versions of the Food Scanner app. Similarly, the Health Heroes app, which aimed to manage healthy portion sizes and a balanced diet in children, was also developed through the guidance of the BCW (15). Twenty-one BCTs were identified, of which six are present within the Food Scanner app. These included “instruction on how to perform the behavior”, “feedback on behavior”, “goal setting”, “prompts/cues”, “information about social and environmental consequences” (updated version only), and “social support (unspecified)”. Results on intervention effectiveness have not yet been published so conclusions regarding effective BCTs cannot be assumed.

Existing research has identified a number of effective BCTs in interventions of childhood obesity prevention that have not been implemented within the Food Scanner app. Guidance has recently been published on the use of suitable BCTs for interventions which support families with primary school-aged children on a “healthy weight journey”(30). Seven of seventeen (41%) of the recommended BCTs were incorporated in the Food Scanner app including “goal setting (behavior)”, “feedback on behavior”, “social support (unspecified)”, “instruction on how to perform behavior”, “social reward”, “prompts/cues” and “behavioral substitution” (dropped in the updated version). Other suitable BCTs that were recommended but were not present within the Food Scanner app included “problem solving”, “action planning”, “self-monitoring of behavior”, “demonstration of behavior”, “behavioral practice/rehearsal”, “graded tasks”, “restructuring the physical environment”, “behavioral contract”, “information about health consequences” and “framing/reframing”. Further consideration of the inclusion of these BCTs may strengthen the app's effectiveness in improving dietary choices. However, little is currently known whether the inclusion of an exhaustive number of BCTs may have positive or adverse impacts on behavior change given that this will increase app complexity. This may interfere with users' experience of, and engagement with the app (32).

One method to deliver “feedback on behavior” in the updated version of Food Scanner app was to include “woah badges” when high fat/sugar/salt items were scanned. Such feedback messages may produce defensive responses (33) and deter users from engaging with the app. In other work, parents rated a disease-based image as the least acceptable option to promote selection of healthy beverages for their children, possibly due to triggering a negative emotional response (34). Similarly, a meta-analysis showed that threat-inducing messages are less effective in achieving behavior change in comparison to other methods (35). Language tone and content used in food purchasing apps can also impact user engagement. Personalized messages have been found to enhance user experience and message salience (36). Furthermore, the integration of notifications and reminders were also helpful to prompt goal priorities. When carrying out app updates, it is therefore important to consider the delivery of BCTs in an engaging format. This will encourage users to engage with the app for the minimum time necessary to gain sufficient exposure to BCTs that could lead to potential behavior change (37).

This research contributes to the growing body of literature concerning the use of effective BCTs in dietary app-based interventions for primary school-aged children and offers a unique insight into how BCT content evolves with app updates and maintenance. However, there are some limitations. Firstly, there was minimal information available concerning the design and content development of the app. For example, it was unclear whether the app was designed according to behavior change theory. This information would have enabled the coders to verify the presence of BCTs and flag any shortcomings in BCT delivery. Secondly, only the BCT content of accessible features of the app could be mapped; there may have been more BCTs present but the features in which they were delivered were not accessed. This happened on at least one occasion; despite the use of the Food Scanner, and purposely scanning healthy products, the “Good Choice badge” feature could not be accessed. Thirdly, coding the outdated version of the app was not fully independent. The second coder used secondary online research due to a major app update leading to the unavailability of v1.6. Despite this, there was a high inter-rater reliability between coders when mapping the outdated version of the app. Fourthly, there is no standardized guidance on identification of near misses. The current study used general guidance from the online training, however it is possible that other near misses were present but overlooked. Given that the identification of near misses could improve future revisions of intervention content, a standardized process for their identification ought to be developed or potentially incorporated within existing BCT coding frameworks. This will highlight missed opportunities of BCT inclusion which may strengthen app development and app effectiveness. Finally, no formal comparison of BCTs was made between differences in dietary choices during the mapping process. More extensive evaluation of the BCT content could compare the use of BCTs between food groups. However, a comprehensive table of BCTs alongside direct examples from the app has been provided within Supplementary Table 1 where it is apparent which food group has been targeted within BCT use.

The Change4Life Food Scanner app has currently not been evaluated for effectiveness in improving dietary choices. To advance the evidence-base around the use of effective BCTs, an evaluation of the app is necessary to verify the results of this current research. A pilot and feasibility trial is currently being undertaken to investigate whether the app is effective in reducing children's sugar consumption over a 3-month period (38). There is also evidence to suggest that multicomponent interventions, whereby the use of a health app is part of a more complex intervention, are more effective than stand-alone app interventions (39). Although there is benefit in evaluating the components of complex interventions separately, future research needs to evaluate the Food Scanner within the broader context of the Change4Life campaign, given that the two are intertwined and the app signposts users to further information on the Change4Life webpages. Recent findings have suggested the effectiveness of a Sugar Smart app (an older version of the Food Scanner app), in reducing sugar consumption when evaluated as part of the multicomponent national Change4Life Sugar Smart campaign. However, findings were not maintained at 12 months follow up (40). The BCTs used in the Sugar Smart app are unknown. However, the app was designed to specifically concentrate on sugars only, rather than macronutrients in the diet, and app features were more simplified than the current app version (40). Given that the use of BCTs and design features of the Food Scanner app are currently more advanced, users may have a more favorable experience with the app now than before. In addition, although the current study investigated the presence of BCTs, important consideration is needed regarding intervention fidelity. Intervention fidelity explores the extent to which an intervention is being delivered, received and enacted in the way it was designed to (41). Although all app-based interventions will be delivered similarly, the exposure to BCT content and design features is highly dependent upon users' engagement with the app (42, 43), and consequently app success in changing behavior. As such, all BCTs identified within the Food Scanner may not be received by the user. Incorporating measures of intervention fidelity is an integral part of intervention evaluation and ought to be incorporated in future trials of digital interventions. Currently this is a gap within the mHealth literature and has been an underexplored area of research.

In conclusion, the current research showed the Change4Life Food Scanner app contains several BCTs that have been found to be effective in dietary interventions. The app does not include many BCTs that have previously been found to be effective within family-based interventions promoting a healthy weight. Recommendations to improve the content of the Change4Life Food Scanner app include strengthening the delivery of features, including more potentially effective and recommended BCTs which are from the same or similar domain and ensuring major app updates do not remove potentially effective BCTs. Future randomized controlled trials are needed to investigate the effectiveness of the Food Scanner app in improving healthy dietary behaviors.

## Data Availability Statement

The original contributions presented in the study are included in the article/Supplementary Material, further inquiries can be directed to the corresponding author.

## Author Contributions

SM conceived and managed the project. SM and EM-D conducted the coding and analysis and first drafted the manuscript. All authors critically reviewed the manuscript and reviewed and accepted the final version of the manuscript.

## Funding

This research was funded in whole, or in part, by the Wellcome Trust [108903/B/15/Z]. The Wellcome Trust had no role in the design, analysis or writing of this article.

## Conflict of Interest

The authors declare that the research was conducted in the absence of any commercial or financial relationships that could be construed as a potential conflict of interest.

## Publisher's Note

All claims expressed in this article are solely those of the authors and do not necessarily represent those of their affiliated organizations, or those of the publisher, the editors and the reviewers. Any product that may be evaluated in this article, or claim that may be made by its manufacturer, is not guaranteed or endorsed by the publisher.
