# Supplementary material for: An Assessment of Behavior Change Techniques in Two Versions of a Dietary Mobile Application: The Change4Life Food Scanner
Source: Front Public Health. 2022 Feb 23;10:803152. doi: 10.3389/fpubh.2022.803152 (PMC8904754; doi:10.3389/fpubh.2022.803152)
Supplement: Supplementary file 1 [file Table_1.DOCX]

Supplementary Material

# Supplemental Table 1. Behavior Change Techniques identified in the Change4Life Food Scanner app

| **BCT, definition and Domain** | **Present in Outdated Version** | **Present in Updated Version** | **Evidence** | |
| --- | --- | --- | --- | --- |
|  |  |  | **Outdated Version (v1.6)** | **Updated Version (v2.0)** |
| 1.1 Goal Setting (Behavior)  *Set or agree on a goal defined in terms of the behavior to be achieved* **Domain 1: Goals and Planning** | ✓ | ✓ | 1. When first opening the app, the following is displayed: “Find out what’s in your food and get tips to make your family healthier”, with a barcode scanner feature above. 2. After scanning a food item: “Can you find a healthier snack?” | “Find good choice badges – You’ll see these when you scan healthier food and drinks. How many will you find?”  This feature sets a challenge to find good choice badges and therefore a goal is set to find them. |
| 2.2 Feedback on behavior  *Monitor and provide informative or evaluative feedback on performance of the behavior (e.g. form, frequency, duration, intensity)* **Domain 2: Feedback and Monitoring** | ✓ | ✓ | 1. Visual depiction of sugar cube equivalents; saturated fat is portrayed in slabs; salt is portrayed in sachets  2. Calorie content  3. Sugar, salt and fat content  4. Phrases in response to scans such as: *“Peanut butter has a surprising amount of sat fat and salt. Spread thinly”; “This choice makes a great start to the day. Enjoy it with fresh fruit”; “This is high in sugar. Look for low sugar swaps with more greens”.* 5. Further Feedback from traffic lights: Users can click on a traffic light where further feedback is provided. E.g. “This is high in sat fat. Look for a reduced fat version”; “This is high in sugar. Look for low sugar swaps with more greens”.  6. ‘View previous scans’ feature | Scan history displays 20 previous scans displaying the name and traffic lights for each product scans, allows user to see what they have previously scanned: 1. Visual Depiction of sugar/fat/salt content  2. Calorie information  3. Traffic lights  4. Virtual reality element (for items with orange or red traffic lights there is a virtual reality animation that demonstrates how much sugar/sat fat/salt is in the item) 5. Low badges – these are shown for products with low amounts of sugar/sat fat/salt.  6. Woah badges - “Woah, that’s a lot!” badges are shown when the amount of sugar, sat fat or salt is more than the app can display within the reveal screen: this is 232g of sugar, 175g of sat fat and 50g of salt. 7. Scan feedback per pack, per portion, per 100g (different presentation styles). Tells you how many grams of sugar, sat fat, salt and calories is in each of these. Tells you what the equivalent amount is in sugar cubes, saturated fat slabs and salt sachets. |
| 3.1 Social Support (Unspecified)  *Advise on, arrange, or provide practical help (e.g. from friends, relatives, colleagues, ‘buddies’ or staff) for performance of the behavior* **Domain 3: Social Support** | ✓ | ✓ | 1. Feedback upon scanning: “This choc is high in sugar and fat! Can you find a healthier snack?”; “Ek, this breakfast choice contains lots of sugar, saturated fat and salt”; “Sugar Alert – look at that sugar, we should have less than 7 cubes of sugar a day”; “sat fat find – look at all those grams of sat fat, we should have less than 28 a day”; “Woohoo! This choice makes a great start to the day. Enjoy it with fresh fruit”.  2. Feature of the link to more ideas (ideas of healthier alternatives, prompt to sub a high salt/sugar/fat item for a healthier) 3. Feature of the link to further information on traffic lights | 1.Refer user to external resources for extra information and support on healthy eating: links to “more ideas for healthy eating”; “Change4life website”. |
| 4.1 Instruction on how to perform behavior  *Advise or agree on how to perform the behavior (includes ‘Skills training’)* **Domain 4: Shaping Knowledge** | ✓ | ✓ | 1. On the instruction section of the app, ‘scan your food and drink and find out what’s inside’ (instruction on how to use the app) 2. Feedback Upon Scanning: “Peanut butter has a surprising amount of sat fat and salt. Spread thinly” (how to consume peanut butter in a healthier way) | Instruction is given in the ‘more info’ section of the app concerning how to use the app:  1. “What’s inside? Scan the barcode to see what’s inside your food and drink” 2. “Let them loose: See the sugar, saturated fat and salt inside your food and drink come to life” 3. “Find good choice badges: You’ll see these when you scan healthier food and drinks. How many will you find?”. It also explains how to use the traffic lights and the meaning of the badges.  4. How to use this app feature 5. “Scan” - “Scan a barcode” |
| 5.2 Salience of consequences *Use methods specifically designed to emphasise the consequences of performing the behavior with the aim of making them more memorable (goes beyond informing about consequences)* **Domain 5: Natural Consequences** | ✓ | ✓ | 1. Once items are scanned, feedback is provided in a number of different ways: Visually – sugar is portrayed in quantity of sugar cube equivalents; saturated fat is portrayed in slabs; salt is portrayed in sachets. This is a memorable way of showing how much sugar/sat fat/salt is in the item and allows the user to visualise the content and understand the content in terms that are more relevant to them e.g. sachets of salt rather than grams of salt. | Use of method specifically designed to emphasise consequences making them more memorable (e.g. animations and imagery presented in relatable terms; rather than just grams of sugar the equivalent is presented as cubes of sugar).  1. Scan feedback: tells you what the equivalent amount is in sugar cubes, saturated fat slabs and salt sachets  2. Let them loose, How much is that: -Animations of sugar cubes/sat fat slabs/salt sachets attacking or overwhelming green person -Image of blue figure dragging bucket, tips it to release the sugar cubes, they start moving and surround him. He falls on the ground and the bucket drops on his head. -Image of blue figure leaning on closed umbrella. Looks up surprised and opens umbrella up, to then see loads of slabs of fat falling from the sky. He then looks at them whilst they’re on the floor in shock/disgust. -Blue man running frantically away from a load of sugar cubes or sachets of salt in which he slips and falls on his back. The sugar cubes/sachets catch up. Salt sachets start pouring out all the salt content beside him, whilst he is lying unconscious on his back with an upside down mouth/frown. -Blue man leaning on umbrella and starts raining salt on him. Looks at the puddle of salt in shock/disgust/unhappy. |
| 5.3 Information about social and environmental consequences *Provide information (e.g. written, verbal, visual) about social and environmental consequences of performing the behavior* **Domain 5: Natural Consequences** | X | ✓ | X | Generalised nutritional information given on the item that is scanned, information is ‘unspecified’ and applicable to all  Examples include: 1.Virtual Reality display of sugar/sat fat/salt content 2. Visual display of sugar/sat fat/salt content |
| 5.6 Information about emotional consequences *Provide information (e.g. written, verbal, visual) about emotional consequences of performing the behavior Note: consequences can be related to emotional health disorders (e.g. depression, anxiety) and/or states of mind (e.g. low mood, stress)* **Domain 5: Natural Consequences** | X | ✓ | X | 1. Virtual Reality display of content:  -Image of blue figure leaning on closed umbrella. Looks up surprised and opens umbrella up, to then see loads of slabs of fat falling from the sky. He then looks at them whilst they’re on the floor in shock/disgust. -Blue man running frantically away from a load of sugar cubes or sachets of salt in which he slips and falls on his back. The sugar cubes/sachets catch up. Salt sachets start pouring out all the salt content beside him, whilst he is lying unconscious (?) on his back with an upside down mouth/frown. -Blue man leaning on umbrella and starts raining salt on him. Looks at the puddle of salt in shock/disgust/unhappy. 2. “This product contains naturally occurring sugars. You don’t need to worry about the sugar in plain milks, as this isn’t added sugar” |
| 7.1 Prompts/Cues  *Introduce or define environmental or social stimulus with the purpose of prompting or cueing the behavior. The prompt or cue would normally occur at the time or place of performance* **Domain 7: Associations** | ✓ | ✓ | 1. Low Badges 2. Sugar/fat/salt alerts with text: ‘sugar alert’ *Eek! This cereal is high in sugar and contains a surprising amount of salt!* | 1. ‘Woah that’s a lot’ badge: appears when there is too much sugar/sat fat/salt content in the food to be able to display on the screen i.e. food with very high content. Badge is red, designed like a stop road sign and surrounded by sugar cubes/fat slabs/salt sachets with angry faces 2. Low badges: badges are awarded when an item with a low sugar/sat fat/salt content is scanned, reinforces successfully finding and scanning a ‘green’ item 3. Traffic lights |
| 8.2 Behavioral Substitution  *Prompt Substitution of the unwanted or neutral behavior* **Domain 8: Repetition and Substitution** | ✓ | X | 1. Feedback is provided regarding the item scanned, with messages pertaining specifically to the high amount of sugar/sat fat/salt within the product: - “This choc is high in sugar and fat! Can you find a healthier snack?” Prompt to substitute for a healthier snack (i.e. unwanted behavior for wanted behavior) - “This is high in sugar. Look for low sugar swaps with more greens” 2. Feature of the link to more ideas (ideas of healthier alternatives, prompt to substitute a high salt/sugar/fat item for a healthier alternative) | X |
| 9.1 Credible Source *Present verbal or visual communication from a credible source in favour of or against the behavior* **Domain 9: Comparison of outcomes** | ✓ | ✓ | 1.Delivery of intervention/app is by PHE in general, who are a credible source, under the “Change4Life” campaign | 1. Delivery of intervention/app is by PHE in general, who are a credible source, under the “Change4Life” campaign |
| 10.4 Social reward *Arrange verbal or non-verbal reward if and only if there has been effort and/or progress in performing the behavior (includes ‘Positive reinforcement’)* **Domain 10: Reward and Threat** | X | ✓ | X | 1. Low Badges: Badges are awarded when an item with a low sugar/sat fat/salt content is scanned; reinforces successfully finding and scanning a ‘green’ item 2. ‘High five, let’s celebrate’, celebration animation: upon scanning an all green item the screen comes up with a ‘happy’ green person and a message that reads “High-Five, Go go green! This is low in sugar, sat fat and salt. Go go green!”  3. Let’s celebrate feature: 3D Feature of green man on a podium dancing and celebrating with confetti under a banner that reads ‘high-five, go go green!’ with images of green man celebrating and offering high fives. |
| 10.5 Social Incentive *Inform that a verbal or non-verbal reward will be delivered if and only if there has been effort and/or progress in performing the behavior*  **Domain 10: Reward and Threat** | X | ✓ | X | 4. ‘Good Choice’ badges feature: the feature of the thumbs up on the badge indicating a positive action (social praise for finding good choice item). User is informed that if effort is put in to find good choice options and then scan them, they will find a good choice badge. |
